# Metabolic Syndrome Fuels Genomic Instability? Insights from a Pilot Study on Colorectal Cancer

**DOI:** 10.3390/cancers17223682

**Published:** 2025-11-18

**Authors:** Salvatore Pezzino, Maria Cristina Scuderi, Ornella Coco, Tonia Luca, Gaetano Magro, Mariacarla Castorina, Stefano Puleo, Sergio Castorina

**Affiliations:** 1 Department of Medicine and Surgery, University of Enna “Kore”, 94100 Enna, Italy; salvatore.pezzino@unikore.it; 2 Mediterranean Foundation “GB Morgagni”, 95125 Catania, Italytluca@unict.it (T.L.);; 3 Department of Medical, Surgical Sciences and Advanced Technologies “G.F. Ingrassia”, University of Catania, 95123 Catania, Italy; gmagro@unict.it

**Keywords:** colorectal cancer, metabolic syndrome, microsatellite instability, pathogenesis, precision oncology

## Abstract

Background/Objectives: Metabolic syndrome (MS) impacts 25% of the adult population worldwide and elevates the risk of colorectal cancer by 40%. Microsatellite instability (MSI) resulting from impaired DNA mismatch repair serves as a critical biomarker for selecting patients for immunotherapy. Methods: This single-center pilot study examined the correlations between MS and MSI in 157 individuals with surgically treated colorectal cancer. Patients were categorized according to the International Diabetes Federation Metabolic Syndrome criteria. The MSI status was assessed using immunohistochemical investigation of mismatch repair proteins. The statistical analysis encompassed chi-square tests and the computation of odds ratios. Results: Patients with MS exhibited a substantially greater prevalence of MSI compared to controls (15.5% vs. 9.8%, *p* < 0.05) corresponding to a 1.63-fold increase in odds. The co-occurrence of MSI and hepatic steatosis displayed a strong association within the MS group (OR: 5.81), indicating a 2.6-fold increased prevalence relative to controls. Conclusions: This pilot investigation offers initial evidence associating MS with a heightened frequency of MSI in colorectal cancer. The strong association with hepatic steatosis indicates common metabolic-genomic pathways. The findings advocate for the incorporation of metabolic assessment into precision oncology for the selection of immunotherapy, necessitating multicenter validation studies.

## Simple Summary

Metabolic syndrome significantly increases colorectal cancer risk. This pilot study examined the association between metabolic syndrome and microsatellite instability, a key biomarker for immunotherapy eligibility. Patients with metabolic syndrome showed markedly higher microsatellite instability prevalence compared to controls, with the strongest association observed when hepatic steatosis was present. These findings suggest shared metabolic-genomic pathways and support incorporating metabolic assessment into precision oncology strategies for optimized immunotherapy selection.

## 1. Introduction

Colorectal cancer (CRC) is a major challenge in contemporary oncology, being the third most prevalent cancer globally and a primary contributor to cancer-related deaths [1,2]. The relationship between metabolic disorders and cancer development is a significant focus of research, especially in light of the global prevalence of metabolic syndrome (MS) [3,4,5,6]. Metabolic abnormalities can affect cellular growth and survival pathways through a variety of mechanisms. Elevated glucose levels impair nucleotide excision repair processes, resulting in the buildup of DNA glycation adducts and increased strand breaks [7,8]. Hyperglycemia increases the activity of 2-ketoglutarate-dependent prolyl hydroxylase, leading to reduced transcription of DNA repair genes via hypoxia-inducible factor-1α [7,8]. Furthermore, metabolic stress inhibits the NAD^+^/NADH ratio and non-homologous end-joining repair, resulting in ongoing DNA damage signaling [9]. Carbohydrate-induced metabolic disturbance triggers senescence cascades by reducing DNA repair ability, resulting in inflammatory phenotypes and tissue malfunction [9]. Furthermore, metabolic syndrome-associated hyperinsulinemia and insulin resistance promote genomic instability via increased mTOR signaling and cellular proliferation, creating conditions favorable to the development of MSI-high malignancies [10,11]. The molecular pathogenesis of CRC encompasses a range of genetic and epigenetic modifications, with microsatellite instability (MSI) identified as a significant molecular marker [6,12,13,14]. MSI, arising from a defective DNA mismatch repair (MMR) system, carries important implications for prognosis and treatment strategies [15,16,17]. The chronic inflammatory condition linked to MS, marked by increased pro-inflammatory cytokines and modified adipokine profiles, may foster a microenvironment that promotes carcinogenesis and particular molecular changes [18,19,20,21]. Recent epidemiological studies indicate that patients with MS exhibit a 2–3 fold increased risk of developing CRC in comparison to those without MS [6,22]. This association is biologically plausible due to several potential mechanisms. Chronic inflammation linked to MS may affect DNA repair mechanisms, particularly the MMR system that maintains microsatellite stability [6,23,24]. Metabolic alterations may influence the epigenetic regulation of MMR genes, potentially resulting in an increased frequency of microsatellite instability [25,26]. The significance of insulin resistance and hyperinsulinemia, key characteristics of MS, in fostering genomic instability has gained recognition. Metabolic disturbances can influence cellular growth and survival pathways, potentially establishing conditions conducive to the development of MSI-high tumors [10,11,27,28]. This pilot study examines the relationship between metabolic syndrome and microsatellite instability in colorectal cancer patients, with the goal of identifying potential clinical and therapeutic implications.

## 2. Materials and Methods

### 2.1. Study Design and Setting

This retrospective, single-center pilot study was conducted by examining the medical records of patients operated between October 2023 and December 2024 at the General Surgery Unit of Azienda Policlinico G.B. Morgagni in Catania, Italy. The study aimed to evaluate the association between metabolic syndrome (MS) and microsatellite instability (MSI) in colorectal cancer, with secondary objectives assessing the impact of metabolic parameters on MSI status and identifying patient subgroups potentially eligible for targeted anti-PD1/PD-L1 immunotherapy. Histopathological examinations, including MSI testing, were performed by a dedicated gastrointestinal pathologist using standardized protocols. Sample size determination followed pilot study conventions, with *n* = 157 based on feasibility considerations rather than formal power calculations. Consistent with the nature of pilot studies, the sample size was not calculated to achieve 80% statistical power, but was determined based on feasibility criteria to inform the design of future large-scale investigations. Eligible participants included adults (≥18 years) with histologically confirmed colorectal adenocarcinoma undergoing curative-intent surgery. Exclusion criteria encompassed hereditary cancer syndromes (e.g., Lynch syndrome), prior neoadjuvant therapy, metastatic disease at diagnosis, and insufficient clinical or laboratory data. Patients were stratified into two groups:

- MS group: Presence of ≥3 International Diabetes Federation (IDF) criteria [29,30].
- Control group: Absence of MS.

Hepatic steatosis was defined and diagnosed using the following criteria: Imaging-based assessment (ultrasound or CT) demonstrating hepatic fat accumulation ≥5% of liver weight.

Data obtained from medical records and imaging reports encompassed: (1) the presence or absence of microsatellite instability assessed via mismatch repair protein immunohistochemistry; (2) the presence or absence of hepatic steatosis evaluated through radiological assessment; and (3) metabolic syndrome status characterized by the presence of ≥3 International Diabetes Federation (IDF) criteria (central obesity, elevated blood pressure, elevated fasting glucose, reduced HDL-cholesterol, and elevated triglycerides) in both patient and control cohorts.

### 2.2. Microsatellite Instability Assessment

Microsatellite instability testing and immunostaining for DNA mismatch repair proteins were performed. Tissue tumor samples were routinely fixed for 24 h in 4% neutral buffered formalin. After paraffin embedding, tumor specimens were cut into 5-μm sections and stained routinely with hematoxylin and eosin (H&E) for histological diagnosis. One block of formalin-fixed, paraffin-embedded tumor tissue was selected per case. Sections were cut and stained by the immunohistochemical method to assess the abnormalities of four MMR proteins (MLH1, MSH2, MSH6 and PMS2) using anti-MLH1, MSH2, MSH6 and anti-PMS2 (mouse monoclonal antibodies from Ventana). Immunostaining procedures were performed using VENTANA detection kits and BenchMark IHC/ISH instruments. The peroxidase reaction was developed using diaminobenzidine tetrachloride as chromogen. Tumor cells were evaluated as negative for protein expression only if they lacked staining in a sample containing internal positive controls (healthy colonocytes and stroma cells). If no immunoreactivity was detected in healthy tissue the results were considered ambiguous. In this study, we employed immunohistochemical assessment of MMR protein expression as a first line marker for MSI status, following established clinical practice guidelines. The relationship between these approaches is well-established: loss of MMR protein expression detected by IHC demonstrates high concordance with MSI-high status determined by molecular methods [31,32,33]. Specifically, tumors showing loss of expression of one or more MMR proteins typically exhibit MSI when tested by PCR-based assays or next-generation sequencing [31,32,33]. For clinical and research purposes, the WHO Classification of Tumours of the Digestive System and NCCN guidelines recognize IHC for MMR proteins as a valid first-line screening approach, with molecular testing reserved for equivocal cases or when discordance is suspected [34]. In our cohort, cases were classified as MSI (dMMR) when loss of nuclear expression of at least one MMR protein was observed in tumor cells with preserved expression in internal controls.

### 2.3. Statistical Analysis

Statistical analysis was conducted using GraphPad Prism version 10.0 (GraphPad Software, San Diego, CA, USA). Categorical variables were expressed as frequencies and percentages. Odds ratios (OR) with 95% confidence intervals were calculated to estimate the relative risk of MSI in the metabolic syndrome group compared to controls. Pearson’s chi-square test was used for group comparisons, with Fisher’s exact test applied when expected frequencies were <5. Statistical significance was set at *p* < 0.05. Given the pilot nature of this study, the sample size (*n* = 157) was not dimensioned to achieve 80% statistical power, but was determined based on feasibility considerations to inform the design of future multicenter studies.

## 3. Results

The study analyzed 157 colorectal cancer patients stratified by metabolic syndrome status and genomic instability features (Table 1).

Table 1 summarizes the absolute number of patients with MSI and those with both MSI and hepatic steatosis in the control group (without metabolic syndrome) and in the group with metabolic syndrome. The odds ratio for MSI, comparing the metabolic syndrome group to the control group, is 1.63. This indicates that patients with metabolic syndrome have 1.63-fold higher odds of presenting MSI compared to controls. For the co-occurrence of MSI and hepatic steatosis, the odds ratio is 5.81. This means that the odds of having both MSI and hepatic steatosis are nearly six times higher in patients with metabolic syndrome than in those without metabolic syndrome.

### 3.1. Prevalence of Microsatellite Instability in Metabolic Syndrome

**Figure 1:**
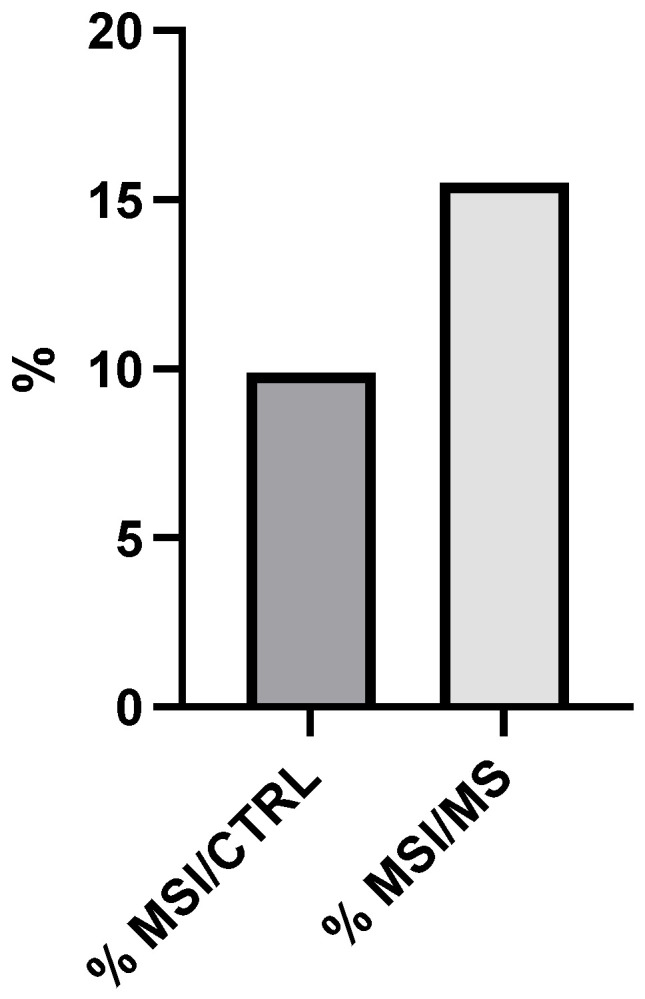
Microsatellite instability (MSI) prevalence in metabolic syndrome (MS) patients vs. control groups (CTRL). Comparison of MSI frequency between MS (15.5%) and CTRL (9.8%), showing a 1.58-fold relative increase (*p* < 0.05, chi-square test).

Figure 1 presents a quantitative comparison of MSI prevalence between the control group (% MSI/CTRL) and the metabolic syndrome group (% MSI/MS). The control cohort exhibits a 9.8% prevalence of microsatellite instability, as indicated by the darker gray bar on the left side of the graph. In contrast, the metabolic syndrome cohort demonstrates a markedly higher MSI prevalence of 15.5%, represented by the lighter gray bar on the right. The vertical axis denotes percentage values ranging from 0 to 20%. This differential represents a 1.58-fold (58%) increase in MSI prevalence in patients with metabolic syndrome compared to control subjects. Statistical analysis confirms the significance of this difference (*p* < 0.05), indicating a non-random association between metabolic syndrome and increased microsatellite instability. MSI, characterized by length alterations in repetitive DNA sequences (microsatellites), typically results from deficiencies in the DNA mismatch repair (MMR) system.

### 3.2. Steatotic Manifestations in MSI-Positive Individuals

**Figure 2:**
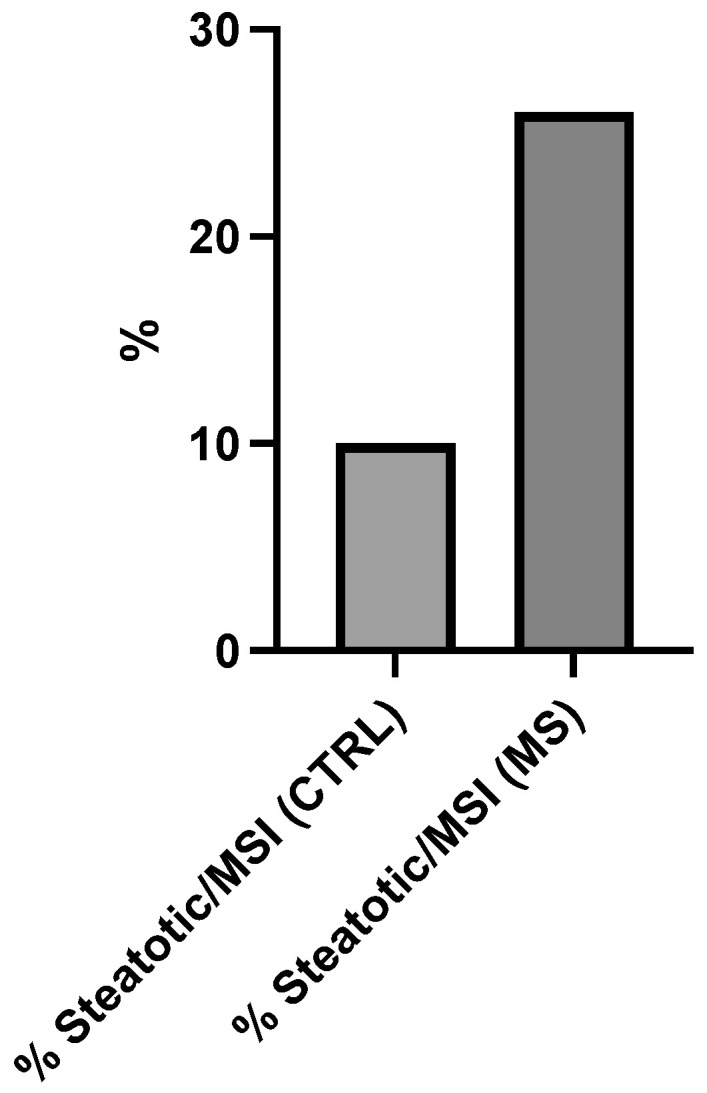
Hepatic steatosis prevalence in microsatellite instability (MSI)-positive colorectal cancer patients stratified by metabolic syndrome (MS) status. X-axis categories represent: % Steatosis/MSI (CTRL) = steatosis prevalence in control group without MS; % Steatosis/MSI (MS) = steatosis prevalence in MS-positive group. Y-axis indicates percentage (%) steatosis prevalence. The 2.6-fold increase in % Steatosis (MS) versus % Steatosis (CTRL) reaches statistical significance (*p* < 0.05, Fisher’s exact test).

Figure 2 further expands the analysis by examining the prevalence of steatotic conditions specifically within MSI-positive individuals from both groups. The graph depicts a striking disparity in steatosis rates between MSI-positive control subjects (% Steatotic/MSI (CTRL)) and MSI-positive metabolic syndrome patients (% Steatotic/MSI (MS)). The vertical axis extends from 0 to 30%.

The control group with MSI demonstrates approximately 10% prevalence of steatotic manifestations. In stark contrast, 26% of MSI-positive individuals with metabolic syndrome exhibit steatotic conditions. This represents a 2.6-fold (160%) increase in steatosis prevalence among MSI-positive metabolic syndrome patients compared to MSI-positive controls.

## 4. Discussion

This pilot study represents a preliminary comprehensive examination of the direct association between metabolic syndrome criteria and microsatellite instability status in colorectal cancer, a relationship that has received limited prior attention in the literature. Our novel study indicates a 1.63-fold elevation in the probabilities of microsatellite instability (MSI) in individuals with metabolic syndrome (MS) compared to controls, and a remarkable 5.81-fold increase when coupled with hepatic steatosis. This new connection goes beyond what has been seen in epidemiological studies by tying a group of metabolic problems—not just one metabolic factor—to a unique genomic instability phenotype. This has strong implications for choosing immunotherapy. It is important to note that the absolute MSI prevalence in our MS-positive cohort (15.5%) is consistent with expected prevalence rates in the general colorectal cancer population [17,35]. However, the finding that MS status functions as an independent predictive variable for this prevalence signifies a previously underexplored association. Prior studies have investigated interconnected facets of metabolic dysfunction and genetic instability; however, our comprehensive approach elucidates essential distinctions. The Northern Sweden Health and Disease Study (*n* = 117,687) indicated that suboptimal metabolic health might serve as a universal mechanism for colorectal cancer pathogenesis, influencing various developmental pathways; however, this prospective analysis failed to reveal significant interactions when categorized by MSI status [36]. In contrast, Nakayama et al. (2019) found an inverse relationship in 936 individuals, indicating that type 2 diabetes mellitus was considerably less prevalent in MSI-high colorectal cancer (3.4% vs. 30.2%, *p* = 0.0007) [37]. Our divergent results—showing a positive correlation between comprehensive MS and MSI prevalence—highlight the biological intricacy of metabolic-genomic interactions and affirm that a systematic evaluation of the entire MS phenotype, rather than individual metabolic variables, is crucial for discerning authentic mechanistic convergence between systemic metabolic dysfunction and genomic instability. Recent mechanistic research gives a lot of support to the idea that metabolism affects genomic stability at the biological level. Studies demonstrate that elevated glucose inhibits nucleotide excision repair pathways, leading to accumulation of DNA glycation adducts and increased strand breaks [8]. This occurs because the activity of 2-ketoglutarate-dependent prolyl hydroxylase is increased, which reduces the transcription of DNA repair genes via hypoxia-inducible factor-1α [8]. A full investigation shows that metabolic stress caused by diabetes makes DNA repair less effective through a number of different pathways [9]. High hyperglycemia messes with the NAD+/NADH ratio and non-homologous end-joining repair, which causes DNA damage signaling to stay on. Recent research shows that carbohydrates start senescence cascades by making it harder for DNA to be repaired. This leads to inflammatory phenotypes and tissue dysfunction [9]. The association between MSI and hepatic steatosis (OR: 5.81) observed in our study reflects mechanistic links through oxidative stress pathways. Research on non-alcoholic fatty liver disease indicates that reactive oxygen species generated by lipids impair genomic stability, with studies revealing significant associations between hepatic oxidative stress and markers of nuclear DNA damage [38]. Recent research indicates that the metabolic-genomic interaction is not confined to liver tissue, demonstrating bidirectional relationships between DNA damage response and cellular metabolism that are essential for preserving genomic integrity [38]. The clinical importance of these findings is rooted in their capacity to improve precision oncology strategies. The KEYNOTE-177 trial established pembrolizumab as the first-line standard of care for MSI-H/dMMR metastatic colorectal cancer, with 5-year follow-up data confirming improved outcomes relative to chemotherapy [39]. The identification of increased MSI prevalence in MS patients (15.5%) indicates that this population may benefit from improved immunotherapy assessment Vargas et al. (2014) identified a gene expression profile related to MS that is significantly associated with clinical outcomes in stage II colorectal cancer patients, thereby confirming the connection between metabolic disorder and colorectal cancer [40]. This metabolic profiling method effectively categorized patients into low-risk and high-risk groups for relapse, highlighting the clinical value of metabolic assessment in the management of colorectal cancer [40]. Recent multi-omics analyses indicate a strong correlation between MS genes and the effectiveness of targeted chemotherapy, especially concerning the mTOR and VEGFR pathways [41]. The findings endorse the incorporation of metabolic stratification into treatment selection algorithms, advancing from conventional molecular markers to a holistic approach of metabolic-genomic profiling [41]. If these findings will be validated in larger trials, metabolic assessment may guide patient classification for MSI-targeted medication. A preliminary framework would categorize patients into three risk levels based on MS status and the presence of hepatic steatosis: low-risk, intermediate-risk, and high-risk. Patients at high risk would necessitate improved MSI testing techniques and increased consideration for checkpoint inhibitor treatment upon confirmation of MSI-high status. Implementation necessitates the incorporation of routine metabolic biomarkers (glucose, triglycerides, HDL, waist circumference, blood pressure) alongside imaging evaluations for hepatic steatosis and standard MSI assessment using immunohistochemistry, with molecular reflex testing when required. This concept could enable precision medicine strategies for colorectal cancer care; nevertheless, prospective validation in larger cohorts is crucial prior to clinical application.

This pilot study’s intrinsic limitations include a small sample size, which limits statistical power, particularly for composite endpoints such as MSI and steatosis analysis. The single-center retrospective methodology may add selection bias, limiting the generalizability of findings to larger colorectal cancer populations. The cross-sectional investigation cannot reveal temporal correlations between metabolic dysregulation and the emergence of chromosomal instability. The findings of this pilot study should be regarded with caution due to the single-center design, small sample size, and potential selection bias. The observational nature prevents causal inferences, and the absence of treatment response data limits its direct clinical usefulness. However, the convergence of observational, mechanistic, and clinical evidence supports the biological validity of identified associations. Large-scale studies, including meta-analyses demonstrating that MS increases colorectal cancer mortality with dose-response relationships based on the number of metabolic risk factors present, provide a supportive epidemiological context [42]. The metabolic-genomic convergence observed in this study corresponds with new therapeutic frameworks. Recent evidence indicates that metabolic interventions may affect genomic stability, with studies showing that modest metabolic enhancements can restore the function of DNA repair proteins [9]. This facilitates the exploration of combinatorial strategies that merge metabolic optimization with immunotherapy in colorectal cancer patients. Clinical trials assessing metabolic interventions, including metformin or GLP-1 agonists in conjunction with checkpoint inhibitors, indicate promising avenues for research [43,44]. The potential for metabolic targeting to improve immune system activation requires systematic investigation in well-designed studies, especially considering the proven efficacy of pembrolizumab in MSI-high colorectal cancer [39].

## 5. Conclusions

This pilot study offers initial evidence for the incorporation of metabolic assessment into colorectal cancer evaluation protocols. The observed associations correspond with known microsatellite instability prevalence patterns, rather than revealing entirely new pathological relationships. However, the biological plausibility of metabolic-genomic interactions and their strong link to hepatic steatosis justify further investigation. The findings add to the increasing evidence that metabolic health serves as a universal mechanism in the development of colorectal cancer, influencing various molecular pathways. The clinical significance is rooted in the potential of metabolic stratification to improve precision oncology methods, especially regarding immunotherapy selection in the context of checkpoint inhibition.

Future multicenter prospective studies with longitudinal follow-up, mechanistic validation, and therapeutic intervention components are necessary to translate these preliminary observations into clinically actionable strategies that enhance patient outcomes through personalized therapeutic approaches. As a pilot investigation, this work provides important feasibility data for larger-scale validation studies while highlighting the complex interplay between metabolic health and genomic stability in colorectal cancer pathogenesis. The results support continued investigation of metabolic targeting as a complementary approach to traditional oncological therapies, with the ultimate goal of improving patient outcomes through integrated metabolic-genomic treatment strategies.

## Figures and Tables

**Table 1:** Comparison of microsatellite instability (MSI) prevalence and MSI + hepatic steatosis co-occurrence between metabolic syndrome patients and controls in colorectal cancer pilot study.

Comparison	Group	With Condition	Without Condition	Odds Ratio (OR)
MSI	Control	9	82	1.63
	Metabolic Syndrome	10	56	
MSI + Hepatic Steatosis	Control	1	90	5.81
	Metabolic Syndrome	4	62	

## Data Availability

The authors confirm that the data supporting the findings of this study are available within the article.

## Abbreviations

The following abbreviations are used in this manuscript:

CRC	Colorectal Cancer
MS	Metabolic Syndrome
MSI	Microsatellite Instability
DNA	Deoxyribonucleic Acid
MMR	Mismatch Repair
NER	Nucleotide Excision Repair
HIF-1α	Hypoxia-Inducible Factor 1-alpha
NHEJ	Non-Homologous End-Joining
NAD+	Nicotinamide Adenine Dinucleotide
NADH	Nicotinamide Adenine Dinucleotide reduced form
mTOR	Mechanistic Target of Rapamycin
IDF	International Diabetes Federation
VEGFR	Vascular Endothelial Growth Factor Receptor
PD-1	Programmed Cell Death Protein 1
PD-L1	Programmed Death-Ligand 1
ISH	In Situ Hybridization
IHC	Immunohistochemistry
H&E	Haematoxylin and Eosin
PCR	Polymerase Chain Reaction
WHO	World Health Organization
NCCN	National Comprehensive Cancer Network
dMMR	Deficient Mismatch Repair
MSI-H	Microsatellite Instability-High
T2DM	Type 2 Diabetes Mellitus
OR	Odds Ratio
CI	Confidence Interval
NAFLD	Non-Alcoholic Fatty Liver Disease
ROS	Reactive Oxygen Species
GLP-1	Glucagon-Like Peptide 1
